# Small noncoding RNA TY2 enhances efferocytosis and improves outcomes in a mouse model of sepsis

**DOI:** 10.1172/jci.insight.196153

**Published:** 2026-01-22

**Authors:** Alessandra Ciullo, Xaviar M. Jones, Hiroaki Komuro, Liang Li, Anh Nguyen, Eduardo Marbán, Ahmed Gamal-Eldin Ibrahim

**Affiliations:** Smidt Heart Institute, Cedars-Sinai Medical Center, Los Angeles, California, USA.

**Keywords:** Cardiology, Inflammation, Macrophages, Noncoding RNAs

## Abstract

TY2 is a drug based on a natural compound with potent anti-inflammatory effects. TY2 improved outcomes in mice with severe bacterial infection.

**To the Editor:** Extracellular vesicles contain bioactive noncoding RNAs (1), including yREX3, a small Y RNA that enhances macrophage efferocytosis by suppressing protein interacting with C kinase 1 (*Pick1*) (2). Here, we report the development of TY2, an exomer (3) bioinspired by yREX3. TY2 includes locked nucleic acids (Figure 1A) and a point mutation (uracil to adenine, yREX3^3’UtoA^) that increased *Pick1* downregulation (Figure 1B) to yield yREX3^3’UtoA^ 3 alternated, renamed TY2 by convention (1) (Supplemental Figure 1A). TY2 was more resistant to RNase degradation (Figure 1C) and enhanced efferocytosis more potently compared with yREX3 (Figure 1D and Supplemental Figure 1B). Rats fed (4) TY2 or yREX3 (3.25 ng/g) after reperfusion had smaller infarcts by histology or circulating cardiac troponin I 48 hours after myocardial infarction (MI) (Figure 1, E–G). Thus, relative to yREX3, TY2 is more stable and at least bioequivalent. To test TY2 in sepsis, we induced cecal ligation and puncture (CLP) in 6-week-old C57BL/6 mice (5) (Figure 1H). Seven days after ligation, mice receiving TY2 showed improved survival (Figure 1I) and greater weight loss, largely due to reduced ascites (Supplemental Figure 1, C and D). Spleen weight was significantly reduced in TY2-treated mice compared with sham mice, indicating reduced inflammation (Supplemental Figure 1, E and F). Serum lactate and aspartate aminotransferase (6) were also attenuated by TY2 (Supplemental 1, G and H). Blood glucose levels decreased in all septic animals compared with sham mice (6) (Supplemental Figure 1I). Echocardiography 1 day after CLP (before treatment) and at endpoint (day 7) revealed reduced cardiac dysfunction (Figure 1J and Supplemental Figure 1J) and remodeling (Figure 1K) with TY2 compared with vehicle. Bacterial counts at endpoint were lower in hearts and lungs of TY2-treated mice (cf. vehicle; Figure 1, L and M). In vitro, macrophages from bone marrow take up inactivated *E. coli* particles conjugated with a fluorophore as a reporter of efferocytosis. Exposure to TY2 increased efferocytosis relative to vehicle (Figure 1, N and O; cf. Supplemental Figure 1, B and D); the effect was independent of animal sex (Supplemental Figure 2, A and B) and time dependent (Supplemental Figure 2C). In vivo, SMAD3 phosphorylation was increased in hearts from CLP mice exposed to TY2, consistent with increased efferocytotic activity (Figure 1, P and Q) and the protective role of PSMAD3 in mitigating inflammation in sepsis (7). Indeed, mice receiving TY2 showed decreased levels of inflammatory cytokines *Tnfa*, *Il1b*, and Il10; a trend for a decrease in *Il6* in heart samples; and, in the lungs, decreased *Il1b* (Supplemental Figure 1K), consistent with less severe sepsis (6). Thus, TY2 mimics yREX3 mechanistically while being more stable, making it worth testing in settings such as sepsis where enhanced efferocytosis may be beneficial.

For detailed methods, information regarding sex as a biological variable, statistics, study approval, author contributions, data availability statement, and acknowledgments, see the supplemental materials.

## Funding support

This work is the result of NIH funding, in whole or in part, and is subject to the NIH Public Access Policy. Through acceptance of this federal funding, the NIH has been given a right to make the work publicly available in PubMed Central.

National Heart, Lung, and Blood Institute (R01 HL142579 to AGEI, R01 HL175335 to AGEI, and NIH T32 HL116273).
CIRM (TRAN1-15317 to AGEI).
Mark S. Siegel Family Foundation Distinguished Chair of Cedars-Sinai Medical Center to EM.

## Supplementary Material

Supplemental data

Supporting data values

## Figures and Tables

**Figure 1:**
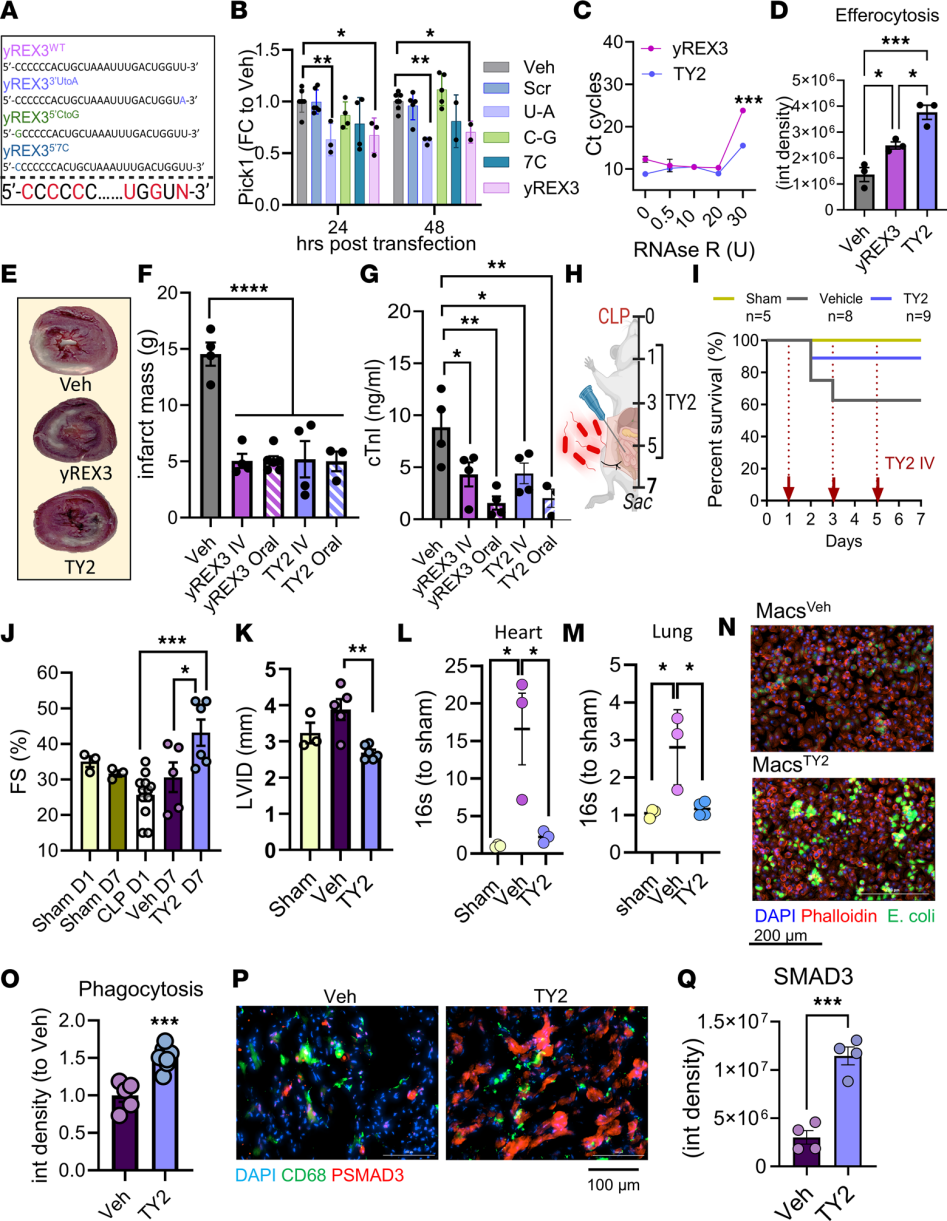
TY2 is protective in a rat model of MI and in a model of cecal ligation and puncture–induced sepsis. (**A**) Schematic of two-step structure activity screening using yREX3 as a template. Locked nucleic acids are shown in red. (**B**) *Pick1* downregulation screening of synthetic candidates. (**C**) Stability of yREX3 and TY2 exposed to increasing concentrations of RNAse R. (**D**) Efferocytosis assay showing uptake of DiD-labeled dead rat cardiomyocytes by rat macrophages at 48 hours. (**E**–**G**) TTC staining showing infarct size (**E** and **F**) and ELISA of circulating cardiac troponin I (cTnI) (**G**) in rats with MI receiving yREX3 or TY2 i.v. or orally by gavage after reperfusion. (**H**) Cecal ligation and puncture–induced (CLP-induced) polymicrobial sepsis model was induced at day 0. Numbers represent days. (**I**) Kaplan-Meier curve showing survival of treatment groups (sham, *n* = 5; vehicle, *n* = 8; TY2, *n* = 9). (**J**) Echocardiographic measurement of cardiac function (fractional shortening [FS] %) at day 1 and day 7. (**K**) Left ventricular internal diameter (LVID; mm). (**L**) Bacterial burden in tissue measured in hearts (**L**) and lungs (**M**) using qPCR of bacterial 16S. (**N** and **O**) Assessment of bacterial (*E*. *coli*) clearance by bone marrow–derived macrophages exposed to vehicle or TY2. Scale bar: 200 μm. (**P** and **Q**) PSMAD3 and CD68 expression in heart sections of mice receiving vehicle or TY2 at 72 hours. Scale bar: 100 μm. Data are presented as mean ± SEM. Dots represent single animals. Statistical analysis of two groups was by Student’s unpaired 2-tailed *t* test, with 95% CI (**C**, **N**, and **Q**) and that of 3 or more groups was by 1-way ANOVA with Tukey’s (**D**, **J**, and **K**) or Dunnett’s (**B**, **F**, **G**, **L**, and **M,** vs. Veh) post test. *P < 0.05, **P < 0.01, ***P < 0.001, and ****P < 0.0001.

## Version 1 01/29/2026

In-Press Preview

## Version 2 03/09/2026

Electronic publication
