# Supplementary material for: Small noncoding RNA TY2 enhances efferocytosis and improves outcomes in a mouse model of sepsis
Source: JCI Insight. 2026 Jan 22;11(5):e196153. doi: 10.1172/jci.insight.196153 (PMC13041665; doi:10.1172/jci.insight.196153)
Supplement: Supplemental data [file jciinsight-11-196153-s014.pdf]

SUPPLEMENTAL MATERIAL

Materials and Methods

Synthesis and formulation of RNA compounds

Synthesis and formulation of RNA compounds yREX3 and its derivatives were synthesized (IDT) and mixed with 3 µl of DharmaFECT® transfection reagent (Horizon Discovery) to a final volume of 100 µl in serum-free media. For *in vitro* applications, a final concentration of 80 nM was used. For *in vivo* applications (intravenous), a similar formulation was prepared to achieve a dose of 2.5 µg/kg. For oral formulation, RNA was mixed with transfection reagent at a concentration of 3.2 µg/kg and then combined with casein/chitosan.

Rat and mouse bone marrow-derived macrophage (BMDM) isolation

Femurs were isolated from 7–10-week-old Wistar–Kyoto rats or 7–10-week-old C57BL/6 mice. Bone marrow was isolated by flushing with PBS and then filtering through a 70-µm mesh. Red blood cells were lysed with ACK buffer (Invitrogen) and then resuspended in IMDM (Gibco) containing 10% FBS, 0.5% gentamycin, and 20 ng/ml M-CSF (eBioscience) for plating. The media was exchanged every 2–3 days until day 5, at which point bone marrow-derived macrophages (BMDMs) were used for in vitro experiments.

RNA purification and qPCR analysis

Total RNA, including small RNAs of approximately 18 nucleotides and greater, was purified using the miRNeasy Mini Kit (Qiagen) according to the manufacturer's protocol. Reverse transcription was performed using the High Capacity RNA to cDNA Kit (Thermo Fisher Scientific) or Taqman® microRNA Reverse Transcription Kit (Applied Biosystems), as per the manufacturer's instructions. Real-time PCR was performed using TaqMan Fast Advanced Master Mix and the appropriate TaqMan Gene Expression Assay (Thermo Fisher Scientific). The reaction was conducted on a QuantStudio™ 12K Flex Real-Time PCR System, with each reaction performed in triplicate. Data were adjusted using hprt1 (Life Technologies). Cycling

conditions followed the TaqMan protocol. Where applicable, the 2–ΔΔCt method was used to determine gene expression fold change.The following primers were used: *Il6* (Mm00446190_m1), *Tnfa* (Mm00443258_m1), *Il1b* (Mm00434228_m1), *Il10* (Mm01288386_m1).

DNA purification and qPCR analysis for 16S rRNA gene

Heart and lung samples were collected, cut into small pieces, and homogenized in 2 ml tubes containing 2.8 mm ceramic beads (Fisherbrand) using a Bead Ruptor 12 (Omni International Inc.). Total DNA was purified using the DNeasy Blood & Tissue Kit (Qiagen) according to the manufacturer’s protocol. DNA was eluted by adding 50 μl of Buffer AE and quantified using a Nanodrop. 100 ng of DNA per well was used for qPCR to detect bacterial DNA using SYBR Green Master Mix (Applied Biosystems). The primer sequence for 16S rRNA is: Forward 5’-AGAGTTTGATCMTGGCTCAG-3’ and Reverse 5’-CTGCTGCSYCCCGTAG-3’.

***In vitro* efferocytosis assay**

To examine macrophage efferocytosis *in vitro*, rat-derived H9C2 cardiomyocytes (ATCC® CRL-1446™) were labeled with DiD (Invitrogen) for 30 minutes at 37°C in the dark, washed, and then starved for 48 hours in serum-free media. The resulting apoptotic cells were counted and added to rat bone marrow-derived macrophage cultures that had been exposed to either vehicle or TY2 for 48 hours. At the appropriate time point (20 minutes), cells were washed twice with PBS, fixed, and stained with Alexa Fluor 488 Phalloidin (Invitrogen, A12379) following the manufacturer’s protocol for immunocytochemical analyses. Images were captured using fluorescence microscopy (Cytation 5, Biotek) and quantified using ImageJ software.

***In vitro* phagocytosis assay**

To examine macrophage phagocytosis *in vitro*, mouse bone marrow-derived macrophage cultures exposed to either vehicle or TY2 for 48 hours were incubated with pHrodo™ Green E. coli BioParticles™ Conjugate for Phagocytosis (Invitrogen) according to the manufacturer’s protocol. At the appropriate time point (20 minutes), cells were washed twice with PBS, fixed,

and stained with Alexa Fluor 594 Phalloidin (Invitrogen, A12381) following the manufacturer's protocol for immunocytochemical analyses. Images were captured using fluorescence microscopy (Cytation 5, Biotek) and quantified using ImageJ software. In alternative, automated fluorescence readings were acquired using a microplate fluorescence reading system (Cytation 5, Biotek) using 509nm-533nm for Excitation-Emission.

Immunohistochemistry

Tissues were embedded in optimal cutting temperature (OCT) compound and frozen in 2-methyl butane pre-cooled in liquid nitrogen and then stored at −80°C until sectioning. Serial sections of the heart were cut at the mid-papillary level in the transverse plane. All sections were cut to between 5 and 6 μm using a cryostat (CM3050S, Leica) and adhered to superfrost microscope slides. Cryosections of the heart were fixed with 4% paraformaldehyde solution (Fisher Scientific, AAJ19943K2) for 10 min, washed with PBS, permeabilized with 0.2% Triton™ X-100 (Millipore Sigma, T8787), and blocked [Protein Block, Dako with 0.05% Saponin (Sigma-Aldrich, S4521)] for 30 min at room temperature. Following the 30 min block, the slides were then incubated overnight with primary antibodies diluted in blocking solution at 4°C. The primary antibodies are as follows: CD68 (1:100, Abcam ab53444), Phospho-SMAD3 (Ser213) (1:100, Abcepta, Cat# AP3250a-ev). After the overnight incubation, the slides were washed with PBS (3 times 5 min) and incubated with the appropriate Alexa Fluor–conjugated secondary antibody (1:200, Invitrogen) for 2 h at room temperature. Following the secondary incubation, the slides were washed with PBS (3 times 10 min) and coverslips were mounted with Fluoroshield with DAPI (Sigma-Aldrich) mounting medium. The slides were imaged using fluorescence microscopy (Cytation 5, BioTek) and quantified using ImageJ software.

Serum collection and marker analysis

Blood was collected from heart of animals at 72hrs, allowed to clot for 10-15 minutes and then centrifuged 2,000g for 10 minutes to collect serum. An aliquot of fresh serum was submitted to

Antech Diagnostics to measure AST and glucose levels; serum lactate levels were measured using the Lactate-Glo™ Assay kit (Promega, #J5021).

Animal Models

Myocardial infarction (MI), oral delivery

All rats were housed in a pathogen-free facility (cage bedding: Sani-Chips, PJ Murphy) with a 14-hour/10-hour light/dark cycle, with food (PicoLab Rodent Diet 20 [no. 5053], LabDiet) and water provided ad libitum. *In vivo* experimental protocols were performed on 7- to 10-week-old female Wistar-Kyoto rats (Charles River Labs, Wilmington, MA). To induce MI, a thoracotomy was performed at the fourth intercostal space to expose the heart under general anesthesia. A 7–0 silk suture was used to ligate the left anterior descending coronary artery, which was removed after 45 minutes to allow for reperfusion. Twenty minutes later, animals received oral delivery via an oral gavage needle (01-290-3B, Fisher Scientific) of vehicle (PBS with DharmaFECT and casein/chitosan), yREX3 (3.2 ng/g animal with DharmaFECT and casein/chitosan), or TY2 (3.2 ng/g animal with DharmaFECT and casein/chitosan).

CLP-induced polymicrobial sepsis model, intravenous injection

In vivo experimental protocols were performed on 7- to 10-week-old C57BL/6 male mice (Jackson Labs). To induce sepsis, mice underwent euthanasia, and a longitudinal midline skin incision was made with a scalpel to enter the peritoneal cavity. After locating the cecum, it was isolated and exteriorized, leaving the remainder of the small and large bowel within the peritoneal cavity. It was critical not to breach or damage the mesenteric blood vessels. The cecum was then ligated at the designated position for the desired severity grade (mid-grade) and perforated by a single through-and-through puncture midway between the ligation and the tip of the cecum in a mesenteric-to-antimesenteric direction. After removing the needle, a small amount of feces from both the mesenteric and antimesenteric penetration holes was extruded to ensure patency. The cecum was then relocated into the abdominal cavity, and the peritoneum and skin were closed with sutures. Finally, animals were resuscitated by injecting prewarmed

normal saline (37°C; 5 ml per 100 g body weight) subcutaneously. Without this step, animals were unable to demonstrate the early, hyperdynamic phase of sepsis. At days 1, 3, and 5, mice received intravenous injections into the retro-orbital venous sinus of vehicle (with DharmaFECT) or TY2 (2.5 ng/g animal, with DharmaFECT). In a shorter protocol (72 hrs), when indicated, animals were subjected to CLP protocol at day 0 and at days 1 and 2 received intravenous injections into the retro-orbital venous sinus of vehicle (with DharmaFECT) or TY2 (2.5 ng/g animal, with DharmaFECT).

Infarct Measurement

TTC staining

Two days post-MI, 10% KCl was injected into the LV to arrest the hearts in diastole. The hearts were harvested, washed in PBS, and cut into 1-mm sections from apex to base, above the infarct zone. Sections were incubated with a 1% solution of 2,3,5-triphenyl-2H-tetrazolium chloride (TTC, Sigma-Aldrich) for 30 minutes at 37°C in the dark and washed with PBS. Sections were then imaged and weighed. The infarcted zones (white) were delineated from viable tissue (red) and analyzed using ImageJ software. Infarct mass was calculated in the tissue sections according to the formula: (infarct area/tot area) / weight (mg).

Cardiac troponin I ELISA

Blood was collected from animals at the study endpoint (from the heart) in EDTA tubes. After being left undisturbed at 4°C for 30 minutes, plasma was obtained following a 15-minute centrifugation at 4000 rpm. Cardiac TnI was quantified using the RAT cardiac troponin-I ELISA kit (Life Diagnostics) according to the manufacturer’s protocol.

Echocardiography

Two-dimensional transthoracic echocardiography was performed (Vevo 3100, Visual Sonics) under light isoflurane anesthesia. Parasternal short-axis B-mode videos were recorded and left ventricular ejection fraction was measured (Visual Sonics v2.0.0 software). Diastolic function was assessed from the apical 4-chamber view by measuring the E/e’ ratio. The E wave (early

filling) was measured by pulse-wave Doppler mode between the tips of the mitral valve; e' was measured with tissue Doppler mode at the septal corner of the mitral annulus. Three separate measurements from each animal were averaged for each parameter.

Sex as a biological variable

In the studies outlined in this manuscript, both male and female animals were used to verify the results. For instance, in the MI studies, female rats were used since female rats have been the model we have used to screen for bioactivity of our small RNA candidates. In the sepsis model, we used male mice since the initial publication that described this model used male mice. Thus, to ensure, consistency of results we relied on male mice. In total TY2 and yREX3 bioactivity was demonstrated to be bioactive in male and female species.

Statistical analysis

Statistical comparisons between groups of two were made using an independent one-tailed or two-tailed independent Student's t-test with a 95% confidence interval. Comparisons made between groups of three or more were made using a one-way analysis of variance with Tukey's post-test to control for multiple comparisons or the Dunnett test.

Study approval

This work does not include data from human subjects research. All animal work performed herein was conducted under approved institutional animal care and use committee (IACUC) protocols.

Data Availability Statement

The data that support the findings of this study are available in the Supporting data values file.

Acknowledgments

We thank Jeanna Huynh for help editing the manuscript.

Author contributions

AC, EM, and AI conceived the idea and wrote the manuscript. AC, LL, HK, XJ, AN performed the experiments and analyzed the data.

Keywords

Macrophages, small RNAs, exomer, noncoding RNA, RNA drugs, efferocytosis, sepsis, inflammation, cardiac, RNA therapeutics.

Supplemental Figure 1.

A. The naturally-occurring, therapeutically bioactive small Y RNA, yREX3 served as bioinspiration for structure-activity optimization studies to develop the lead candidate, TY2. **B.** Efferocytosis assay showing the capacity of TY2-exposed macrophages to uptake DiD-labeled dead rat cardiomyocytes (representative images taken at 48 hours, n=3 biological replicates/group) **C.** Body weights (BW, grams) of animals recorded at day 0 (before the procedure) and at endpoint (day 7). **D.** Enhanced ascites in CLP mice given vehicle compared to those given TY2 **E.** Spleen weights of animals exposed to different treatment regimen. **F.** Distension spleen size in CLP mice given vehicle compared to those given TY2. **G**. Serum lactate levels in CLP-mice receiving vehicle or TY2 compared to sham. **H.** AST levels indicating hepatic dysfunction were upregulated in vehicle-treated animals and their levels decrease in TY2 treatment at 72 hrs. **I.** Levels of glucose in the blood dropped in CLP animals compared to sham. **J**. Echocardiographic measurement of ejection fraction (EF, %) at day 1 after CLP induction and before the first injection and at day 7. **K**. QPCR in heart samples for expression levels of clinically relevant cytokines in vehicle- and TY2-treated mice at day 7 (data presented as fold change compared to sham animals) and in lung samples., data presented as mean ± SEM, with dots representing single animals. Statistical analysis was done using One-Way ANOVA with Tukey's post-test to control for multiple comparisons; *$p<0.05$, **$p<0.01$, ***$p<0.001$, and ****$p<0.0001$.

Supplemental Figure 2.

A, B. Assessment of bacterial (E. coli) clearance by BMDMs exposed to vehicle or TY2 using automated fluorescence readings at different concentration (40nM, 80nM) for 48 hrs in both male (**A**) and female (**B)** animals. **C.** Phagocytotic capacity is time-dependent with a peak at 48 hrs. Data presented as mean ± SEM, with dots representing independent readings. Statistical analysis of two groups was done using a Student's unpaired t test with 95% CI (**C**), or, of three groups, by One-Way ANOVA with Dunnett's post-test (**A, B**); **$p<0.01$, ***$p<0.001$, and ****$p<0.0001$.

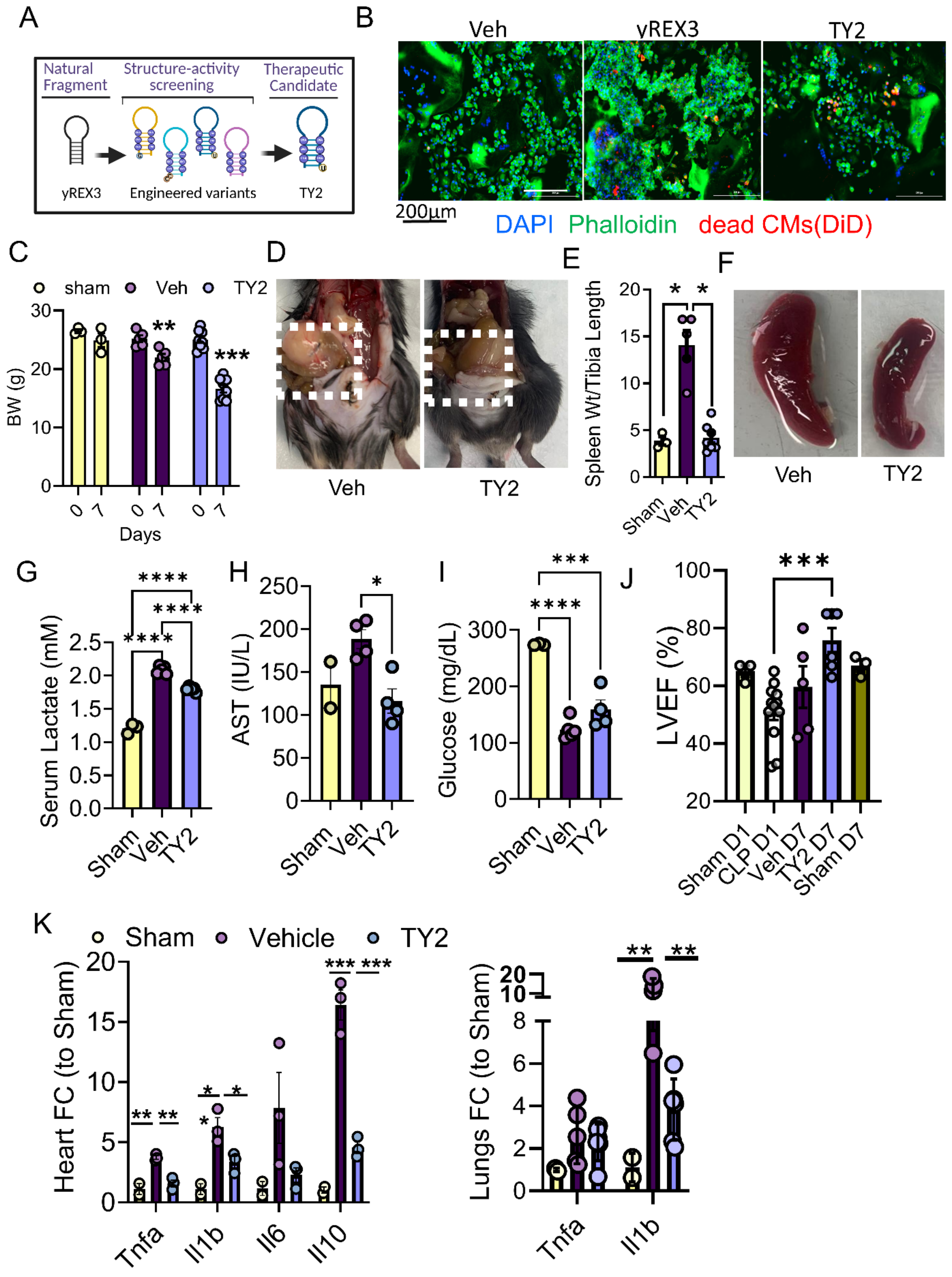

A
Natural Fragment
Structure-activity screening
Therapeutic Candidate
yREX3
Engineered variants
TY2
B
Veh
yREX3
TY2
200μm
DAPI Phalloidin dead CMs(DiD)
C
sham Veh TY2
BW (g)
Days
D
Veh
TY2
E
Spleen Wt/Tibia Length
Sham Veh TY2
F
Veh
TY2
G
Serum Lactate (mM)
Sham Veh TY2
H
AST (IU/L)
Sham Veh TY2
I
Glucose (mg/dL)
Sham Veh TY2
J
LVEF (%)
Sham D1 CLP D1 Veh D7 TY2 D7 Sham D7
K
Sham Vehicle TY2
Heart FC (to Sham)
Tnfa Il1b Il6 Il10
Lungs FC (to Sham)
Tnfa Il1b

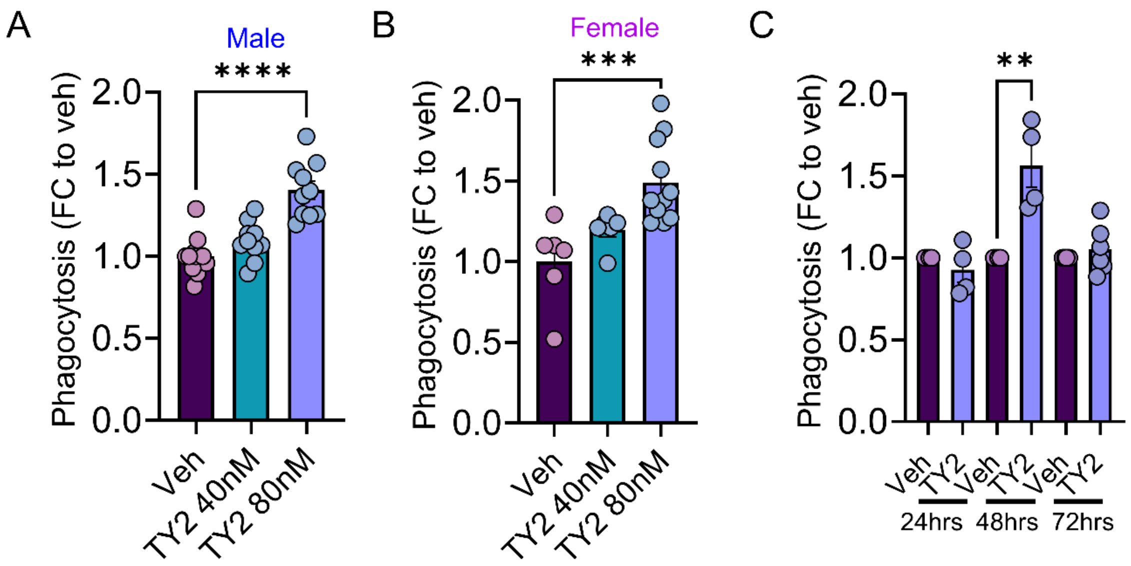
A
Male
****
Phagocytosis (FC to veh)
2.0
1.5
1.0
0.5
0.0
Veh
TY2 40nM
TY2 80nM
B
Female
***
Phagocytosis (FC to veh)
Veh
TY2 40nM
TY2 80nM
C
**
Phagocytosis (FC to veh)
Veh
TY2
24hrs
48hrs
72hrs
